# Regulatory Mechanism of Protein Crotonylation and Its Relationship with Cancer

**DOI:** 10.3390/cells13211812

**Published:** 2024-11-02

**Authors:** Siyi Yang, Xinyi Fan, Wei Yu

**Affiliations:** 1 Institute of Biochemistry, College of Life Sciences and Medicine, Zhejiang Sci-Tech University, Hangzhou 310018, China; ysy1143075794@163.com; 2 Zhejiang Provincial Key Laboratory of Silkworm Bioreactor and Biomedicine, Hangzhou 310018, China; 3 Faculty of Arts and Science, University of Toronto, Toronto, ON M5S 1A1, Canada; xin.fan@mail.utoronto.ca

**Keywords:** protein crotonylation, regulatory mechanisms, cancer

## Abstract

Crotonylation is a recently discovered protein acyl modification that shares many enzymes with acetylation. However, it possesses a distinct regulatory mechanism and biological function due to its unique crotonyl structure. Since the discovery of crotonylation in 2011, numerous crotonylation sites have been identified in both histones and other proteins. In recent studies, crotonylation was found to play a role in various diseases and biological processes. This paper reviews the initial discovery and regulatory mechanisms of crotonylation, including various writer, reader, and eraser proteins. Finally, we emphasize the relationship of dysregulated protein crotonylation with eight common malignancies, including cervical, prostate, liver, and lung cancer, providing new potential therapeutic targets.

## 1. Introduction

Chemical modifications of proteins are known as post-translational modifications (PTMs), which constitute an important epigenetic mechanism that controls protein expression, transcription, development, and cell differentiation [1]. PTMs change the function or localization of proteins by covalently adding functional chemical groups to one or more amino acid residues, thus greatly enriching the proteome [2]. The addition of these functional groups changes the protein’s charge, molecular weight, and conformation, affecting its activity, subcellular localization, and interactions with other proteins [3], thus expanding its range of biological functions. As the only proteinogenic amino acid with an ε-amino group, lysine can undergo a variety of acylation modifications. According to the main properties of the introduced groups, these modifications can be classified into three categories, as shown in Table 1. Among them, acetylation (Kac) [4], propionylation (Kpr) [5], butyrylation (Kbu) [6], crotonylation (Kcr) [7], and benzoylation (Kbz) [8] introduce hydrophobic groups that neutralize the positive charge [9]. By contrast, malonylation (Kmal) [10], succinylation (Ksucc) [11], and glutarylation (Kglu) [12] introduce acidic acyl groups that change the positive into a negative charge. In addition, 2-hydroxyisobutyrylation (Khib) [13], β-hydroxybutyrylation (Kbhb) [14], and lactylation (Kla) [15] introduce polar acyl groups that can form hydrogen bonds with interacting molecules [9]. These chemical modifications can not only change the structure of proteins but also regulate their stability and activity [16]. Dysregulated PTMs may cause cell dysfunction and eventually lead to pathological processes such as carcinogenesis and metastasis.

**Figure 1:**
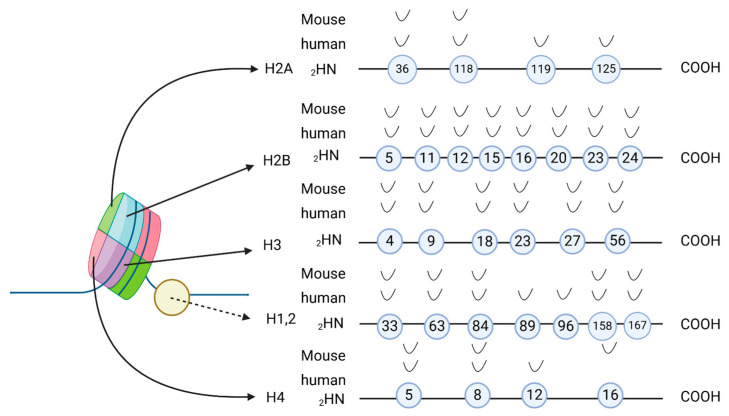
Distribution of crotonylation on histones. The schematic diagram shows the major lysine crotonylation sites on histones H1.2, H2A, H2B, H3, and H4, as well as the differences between human and mouse histones.

In 2011, Tan et al. analyzed 67 new histone modification sites on human chromosomes, leading to the discovery of a novel evolutionarily highly conserved histone modification named histone lysine crotonylation (Kcr) [7]. This discovery has spurred scientists to focus on similar PTMs using integrated mass spectroscopy-based proteomic approaches. This was made possible by the high sensitivity of isoelectric focusing (OFFGEL) and LTQ Orbitrap Velos mass spectrometers, which rely on in vitro propionylation for comprehensive histone PTM analysis. This method achieved high sequence coverage of peptide profiles, and Kcr was identified as a new histone marker. In said study, 28 crotonylation variants were found in humans and 24 in mice, as shown in Figure 1. Histone crotonylation was initially reported as a special marker related to sex determination. In the genomes of human somatic cells and mouse male germ cells, histone Kcr specifically marks the transcription start site (TSS) of active genes and some specific X-linked genes. These genes can escape the inactivation of neutral chromosomes in haploid cells after meiosis [7]. Kcr is introduced by crotonyltransferases that use crotonyl coenzyme A as a substrate. With the rapid development of mass spectrometry (MS) and proteomics, Kcr was subsequently found in various microorganisms, plants, and animals [17,18,19]. For example, Zhu et al. found that Bspf, an effector protein secreted by the type IV secretion system (T4SS) of *Brucella* sp., can regulate host secretion and transport, thus promoting the survival and reproduction of the bacterium. Notably, Bspf was found to exhibit both acetylase and crotonyltransferase activities. It was, therefore, speculated that Bspf may affect the function of host proteins by altering their crotonylation, thus promoting the propagation of *Brucella* [20]. Lin et al. found that Bspf could regulate host cell apoptosis by weakening the crotonylation of p53 and reducing its expression, prolonging the intracellular survival of *Brucella* [21]. Two studies revealed the pathogen-induced changes of protein crotonylation in host cells, providing a new way to further study the mechanism of microbial infection. Crotonylation occurs not only on histone lysines but also on serine 46 of p53 and other non-histone proteins [22]. In 2017, Xu et al. discovered that crotonylation can occur in non-histone proteins [23]. Lung cancer cell line H1299 was used as the starting material to enrich Kcr peptides with a highly specific pan-Kcr antibody. The peptide was identified by high-resolution liquid chromatography-tandem mass spectrometry (LC-MS/MS). A total of 2696 Kcr sites in 1024 proteins were identified. Among them, 40% of the Kcr-modified proteins were located in the cytoplasm, 27% in the nucleus, and 13% in mitochondria. The distribution of Kcr modifications in mouse tissues was also analyzed by immunohistochemistry, and it was found that Kcr was widely present in various tissues of mice, suggesting that it may be involved in many physiological processes.

Over the past decades, the abundant, reversible, and highly regulated lysine acetylation (Kac) has irrefutably become a ‘hot spot’ in the field of epigenetic modification. Kcr shares many of the same regulatory factors and sites with Kac, but unlike Kac, the Kcr modification group contains four carbons. It has a rigid plane conformation due to the carbon–carbon(C-C) π bond, while Kac is tetrahedral and can freely rotate, indicating that Kcr may have more special biological functions [24]. With increasing attention being paid to crotonylation, it has been proved to be involved in physiological processes such as RNA processing, nucleotide metabolism, and chromatin recombination [25]. In addition, Kcr is also associated with many diseases, including infections, spermatogenesis disorders, as well as kidney, cardiovascular, and neurological diseases. However, Kcr appears to have the closest relationship with cancer.

Cancer is characterized by uncontrolled cell growth and spread to other parts of the body. It can originate in virtually any part of the human body, which consists of trillions of cells. Normally, these cells undergo division to grow and multiply into healthy tissues under strict control. As old or damaged cells die off, they will be replaced by new ones. Cancer is a genetic disease caused by gene changes that control cell death, survival, and proliferation. The most prevalent forms of cancer include breast, lung, colon, rectal, and prostate cancer.

This article provides a comprehensive review of the recent studies on the regulatory mechanism of Kcr, particularly its relationship with various cancers, hoping to provide new insights into potential therapeutic targets.

## 2. Mechanism of Action of Crotonylation

**Figure 2:**
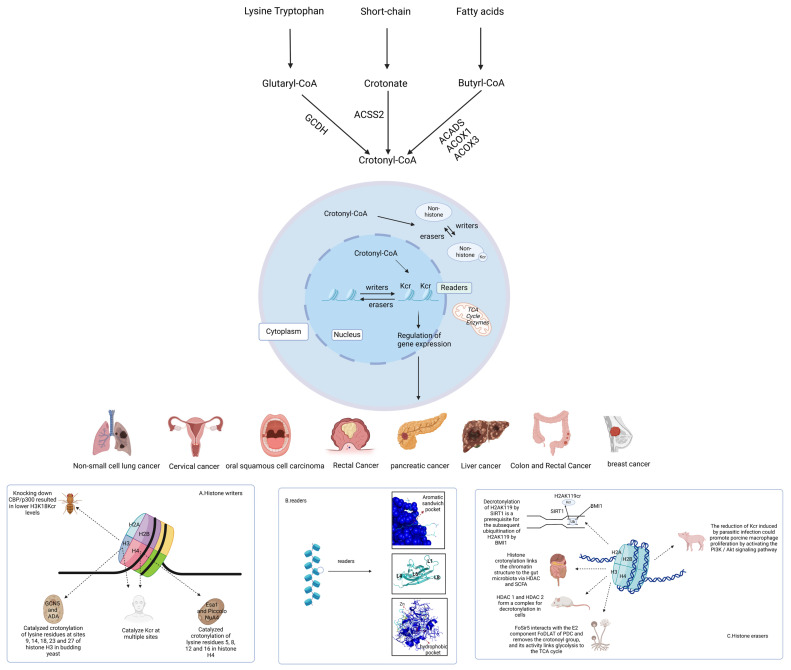
Regulatory mechanism of protein crotonylation. Catabolism of lysine or tryptophan generates crotonyl-CoA, catalyzed by GCDH. Circulating crotonate can be converted into crotonyl-CoA by ACSS2, and fatty acid β-oxidation produces crotonyl-CoA upon oxidation of butyryl-CoA, catalyzed by ACADS, ACOX1, or ACOX3. Protein crotonylation is affected by various writing, reading, and erasing proteins and is closely related to a variety of cancers. (**A**) indicates writing proteins, (**B**) reading proteins, and (**C**) erasing proteins. GCDH: glutaryl-CoA dehydrogenase; ACADS: short-chain specific acyl-CoA dehydrogenases; ACOX1: peroxisomal acyl-coenzyme A oxidese1; ACOX3: peroxisomal acyl-coenzyme A oxidese3; ACSS2: member of the acyl-CoA synthetase short-chain family 2; GCN5: histone acetyltransferase; ADA: adenosine deaminase; HDAC: histone deacetylases; SIRT1: silent information regulator protein 1; SCFA: short-chain fatty acid.

Epigenetic regulation refers to heritable control of gene expression without altering the nucleotide sequence. This includes the regulation of non-coding RNA, as well as covalent modifications of histones and DNA. Histone-modifying proteins include factors that are responsible for adding (writers), recognizing (readers), and removing (erasers) modifications [26]. Kcr is governed by the dynamic equilibrium of enzyme activity between writers and erasers. The study of regulatory factors is crucial for understanding the underlying mechanisms, and the regulatory factors of Kcr are presented in Table 2. Crotonylation is a covalent modification that transfers the crotonyl group onto lysine residues by using crotonyl-CoA as substrate under the action of crotonyltransferase. It usually occurs in gene promoter and enhancer regions [27]. Crotonic acid is a prevalent short-chain fatty acid. Crotonic acid enters cells through free diffusion or MCT-1 vectors, and crotonate enters cells through SMCT-1 vectors and then is converted to crotonyl coenzyme A by acyl-coenzyme A synthetase 2 (ACSS2). Crotonyl coenzyme A is the primary substrate of Kcr writers, but its metabolism is not entirely clear. Butyrate can be converted to butyryl-CoA via the fatty acid β-oxidation pathway and converted to crotonyl-CoA by glutaryl-CoA dehydrogenase (GCDH). The short-chain-specific acyl-CoA dehydrogenases(ACADS) and peroxisomal acyl-coenzyme A oxidese1, 3(ACOX1, ACOX3), which facilitate the conversion of butyryl-CoA to crotonyl-CoA during the process of fatty acid oxidation, have been demonstrated as crucial producers of crotonyl-CoA during endodermal differentiation [28]. In the amino acid metabolism of lysine, hydroxylysine, and tryptophan, glutaryl-CoA dehydrogenase catalyzes glutaryl-CoA oxidation to crotonyl-CoA and CO_2_ [29]. Some scholars speculate that crotonic acid, similar to butyric acid, is a byproduct or an intermediate produced by specific intestinal flora in the body; however, it has not been experimentally proven [30]. Previous studies have found that some protein Kcr sites already exist on mitochondria [31], and the nuclear localization of Pdha1 in the mitochondrial TCA cycle enzyme can provide reaction reads for histone acetylation by promoting the synthesis of acetyl-CoA in the nucleus and providing a new idea for incorporating crotonylation into the nucleus [32]. There are two main ways to manipulate crotonyl levels experimentally. One is to use disodium crotonate, and the other is to obtain sodium crotonate through the reaction of crotonate with sodium hydroxide. Both can upregulate Kcr, and the regulatory modification process is shown in Figure 2. In addition to the intracellular crotonyl CoA concentration, the regulation of protein crotonylation is mainly mediated by relevant writer and eraser enzymes [33]. The level of intracellular crotonylation is maintained by the balance of enzyme activity between writing and erasing proteins.

### 2.1. Writers

They share parts of their regulatory machinery because of the structural similarity between crotonylation and acetylation. Among the enzymes that introduce the modification, also called writers, there are three histone acetyltransferases (HAT) that have also been shown to have histone crotonyltransferase (HCT) activity, including p300/CBP, MYST, and GCN 5. Among them, P300/CBP was the first discovered crotonyltransferase [27] with both HAT and HCT activities [34], whereby p300-catalyzed histone Kcr can directly stimulate transcription. Increasing the concentration of crotonyl-CoA resulted in more crotonylation modification and less acetylation modification, and there was a competitive relationship between them [34]. In vitro studies have demonstrated that the knockdown of p300 expression by siRNA leads to a decrease in the crotonylation level of H3K18 [63], but this activity is significantly lower than its acetyltransferase activity due to the limited aliphatic pocket size for the binding of crotonyl-CoA [64]. Nevertheless, p300/CBP is still considered the dominant histone HCT in mammalian cells [43]. Histone crotonylation was subsequently detected in various eukaryotes, including yeast, but no p300/CBP homologs were identified, suggesting that these organisms maybe contain other HCTs [65]. The MYST protein family, which includes human MOF and its yeast homolog ESA1, can catalyze histone Kcr at multiple sites, including H3K4, H3K9, H3K18, H3K23, H4K8, and H4K12 [36]. In budding yeast, ESA1 is mainly responsible for histone Kcr [36]. However, the in vitro HCT activity of human MOF and yeast ESA1 was relatively weak, indicating that they may function in vivo as components of protein complexes [66]. The reason that GCN5 can catalyze the crotonylation of H3K9, 14, 18, 23, and 27 in budding yeast [36], while ESA1 can catalyzes the crotonylation of H4K5, 8, 12, and 16 [67]^,^ is attributed to the ESA1-Yng 2-Epl 1 (Piccolo NuA4) and GCN5-Ada2-Ada3 (ADA) complexes, respectively. Non-histone nucleophosmin 1 (NPM1) can be strongly crotonylated by CBP and MOF, as well as being moderately crotonylated by p300/CBP-associated factor (PCAF). Moreover, crotonylation of non-histone recombinant DEAD box polypeptide 5 (DDX 5) can only be catalyzed by CBP, and not by other writers [23]. In addition, CBP acts as the crotonyltransferase of ENO1 [68]. Tip60 histone acetyltransferase (TIP 60) mediates Kcr of EB1 at K66 and is involved in mitosis [37]. Lysine acetyltransferase 7 (KAT7) mediates K525 crotonylation of calnexin (CANX) after leucine deprivation [38]. Unfortunately, current research on Kcr writers is based on known HATs, and no new discoveries have been made.

### 2.2. Readers

The so-called readers are proteins that can specifically recognize post-translational modifications. Three Kcr reader families have been discovered, including the bromo-domain structural domain proteins, the YEATS structural domain proteins (Yaf9, ENL, AF9, Taf14, and Sas5), and the double PHD finger (DPF) proteins [35]. Certain subsets of bromodomain proteins, such as BRD9 and TAF1, can recognize Kcr, but their binding affinity for crotonylated peptides is much weaker than for acetylated peptides [40]. In 2016, the YEATS domain was identified as the first effective reader of histone Kcr [41]. Some researchers hypothesized that the YEATS domain may be more receptive to longer and larger acyl groups [65], and it was subsequently demonstrated that it tends to bind longer acyl chains than acetyl groups and has a higher affinity for Kcr [39]. Andrews et al. found that the transcription initiation factor TFIID subunit 14 (Taf14) acts as a reader of histone crotonylation [42]. The YEATS domain of Taf14 binds to H3K9Cr via a π-π-π stacking interaction [24]. Subsequently, various proteins containing the YEATS domain were found to contain selective histone Kcr reading functions, such as YEATS2, which binds to crotonylated proteins through a terminal open aromatic sandwich pocket, which binds weakly to crotonylated H3K9, H3K12, H3K23 Cr, and H4K4, but also very strongly to crotonylated H3K27 [42]. However, the YEATS domain of AF9 closely binds to crotonylated H3K9, H3K18, and H3K27 via a pocked formed by its L1, L4, and L6 loops [39]. In addition, the DPF domain has been recognized as a second reader of histone Kcr. The DPF domain of MYST family members monocytic leukemia zinc finger protein (MOZ) and DPF 2 selectively recognize crotonylated H3K14. The DPF domain produces a hydrophobic “dead-end” pocket that has no aromatic double-stranded residues to accommodate Kcr, but its binding capacity is 4-8 times greater than that of Kac [24]. In addition, the DPF domain of monocytic leukemia zinc finger associated factor (MORF) also showed selectivity for crotonylated H3K14 compared to acetylated H3K14 [69]. This is attributed to the selectivity of histone Kcr readers, but more in-depth research is needed.

### 2.3. Erasers

The erasing protein is known as a decrotonylase, which catalyzes a covalent reaction that removes the crotonylation of lysine. Histone deacetylases (HDACs) are mainly classified into four groups: the Zn^+^ dependent class I (HDAC1, 2, 3, and 8), class II (HDAC4, 5, 6, 7, 9, and 10), the NAD^+^ dependent class III (sirtuins1-7), and class IV (HDAC11). In 2012, histone deacetylase 3 (HDAC3) was first reported to have histone decrotonylase (HDCR) activity [70]. By using fluorescent substrates to systematically screened the activity of eleven tapes of human zinc-dependent lysine deacylases, the authors found that HDAC3 and nuclear receptor corepressor 1 (HDAC3-NCOR 1) exhibited decrotonylase activity [69]. Recent studies have shown that the class I enzymes HDAC1, HDAC2, and HDAC3 can effectively remove crotonyl moieties from modified histones in vitro and may play a role in the formation of complexes in cells, including HDAC1/2, HDAC3/Ncor1, and HDAC1/CoR-EST1/LSD1 [71,72]. In addition, HDAC1/2 has been identified as the dominant HDCR in embryonic stem cells (ESCs), where its genetic deletion resulted in an 85% reduction in total HDCR activity [72]. HDAC2 regulates the level of H2BK12 crotonylation [45]. HDAC1 can hydrolyze the crotonyl modification on H3K18 and, therefore, affects epigenetic regulation in cancer [33]. Subsequently, SIRT1-3 was identified as an additional decrotonylase. Feldman et al. used the H3 peptide containing Lys9 to conduct a comprehensive analysis of the activities of seven mammalian silenced regulatory proteins and found that the HDCR activity of Sirt1 and Sirt2 could catalyze the removal of crotonyl modifications from the histone H3K9 peptide in vitro [49]. In addition, the Kcr of H2AK119 is mediated by SIRT-1 [50]. The de-crotonylation of H2AK119 by SlRT1 is a prerequisite for the subsequent ubiquitination of H2AK119 by BMI1. Bao et al. found that knockdown Sirt3 led to an increase in the related histone Kcr [51]. FoSir5, a direct homolog of human lysine deacetylase SIRT5, interacts with FoDLAT—the E2 component of PDC—and decrotonylates H3K18, leading to the downregulation of the transcription of genes encoding components of the aerobic respiratory pathway [48]^,^ and its activity links glycolysis with the TCA cycle. In addition, the knockdown of SIRT6 was found to induce a significant increase in H3K27 crotonylation [52]. Wei et al. stated that HDAC is the main component of HDCR [73]. Knocking down HDAC1, HDAC2, or HDAC3 in HeLa cells increased of crotonylation and acetylation of histones, and the effect was stronger when HDAC1/3 were both knocked down at the same time. However, selectively knocking down SIRT1 or SIRT3 had no significant effect on overall histone crotonylation, nor did knockdown of SIRT1/3/5, suggesting that class I HDACs may be the dominant HDCRs in mammalian cells [73]. It was also found that SIRT1 and class I HDACs share the same catalytic center, participating in both histone deacetylation and de-crotonylation [73]. However, the target sites of HDAC1 include crotonylated H3K4, H3K9, H3K23, H4K8, H4K12, and H3K18 [73], while those of SIRT1 includes crotonylated H3K9, H4K8, H3K4, H3K18, and H2AK119. Histone crotonylation links chromatin structure to gut microbiota through HDAC and SCFA; de-crotonylation on H2BK12 induced by parasitic infection promotes porcine macrophage proliferation by activating the Pl3K/Akt signaling pathway. Hence, they are histone decrotonylases with different specificities. Similar observations were made in non-histone proteins. For example, when endogenous levels of HDAC3 or HDAC1 were knocked down, the Kcr of NPM1 was enhanced. In addition, a recent study found that HDAC7 affects leucine deprivation-induced autophagy by regulating the Kcr of non-histone protein 14-3-3ϵ at K73 and K78 [47]. In addition, HDAC3 reduced the crotonylation level of non-histone AKT1 during myogenic differentiation [44]. Lamin A was also found to be crotonylated at K265/270, which is regulated by HDAC6 [46]. SIRT2 is involved in the de-crotonylation of ENO1 [35], and SIRT7 induces the K25 de-crotonylation of PHD finger protein 5A (PHF5A) to regulate aging [53]. These studies have shown that HDAC1, HDAC3, HDAC6, HDAC7, SIRT2, and SIRT7 can also act as decrotonylases of non-histone proteins.

### 2.4. Other Influencing Factors

In addition to writers, readers, and erasers, other regulatory factors may also be involved in the regulation of crotonylation. The chromosome domain Y-like transcriptional corepressor (CDYL) is a chromatin reader protein [54]. However, it can also regulate crotonylation of histones because of its crotonyl-CoA hydratase activity, which was found to be associated with transcriptional inhibition [55]. In addition, enzymes that regulate the production and degradation of crotonyl-CoA are also regulators of Kcr, including ACSS2 [58], short-chain enoyl CoA hydratase 1 (ECHS1) [56], glutaryl CoA dehydrogenase (GCDH) [57], short-chain acyl-CoA dehydrogenase (ACADS) [28], as well as acyl-CoA oxidases 1-3 (ACOX1-3) [28,59,61]. Also relevant are various glycolysis rate-limiting enzymes such as phosphoglycerate kinase 1 (PGK1) [62], fructose diphosphate aldolase A (ALDOA) [62], α-enolase (ENO1) [62], and ATP-dependent 6-phosphofructokinase (PFK) [62], among which PGK1 has multiple crotonylation sites. Unlike ACSS2 and CDYL, which directly regulate the synthesis and degradation of crotonyl-CoA, a Nuclear Paraspeckle Assembly Transcript 1 (NEAT1) is a long non-coding RNA. It can recognize and locate p300/CBP, silence the expression of NEAT1, increase the crotonylation of H3K27, and decrease the acetylation of H3K27 [74], thereby promoting disease progression. In prostate cancer cells, bromine domain protein 4 (BRD4) regulates p300 and GCN5 levels to influence histone Kcr [60]. In triple-negative breast cancer, solute carrier family 7 member 2 (SLC7A2) mediates lysine catabolism and promotes histone crotonylation by upregulating ACOX1 [61].

## 3. Protein Crotonylation in Cancer

**Figure 3:**
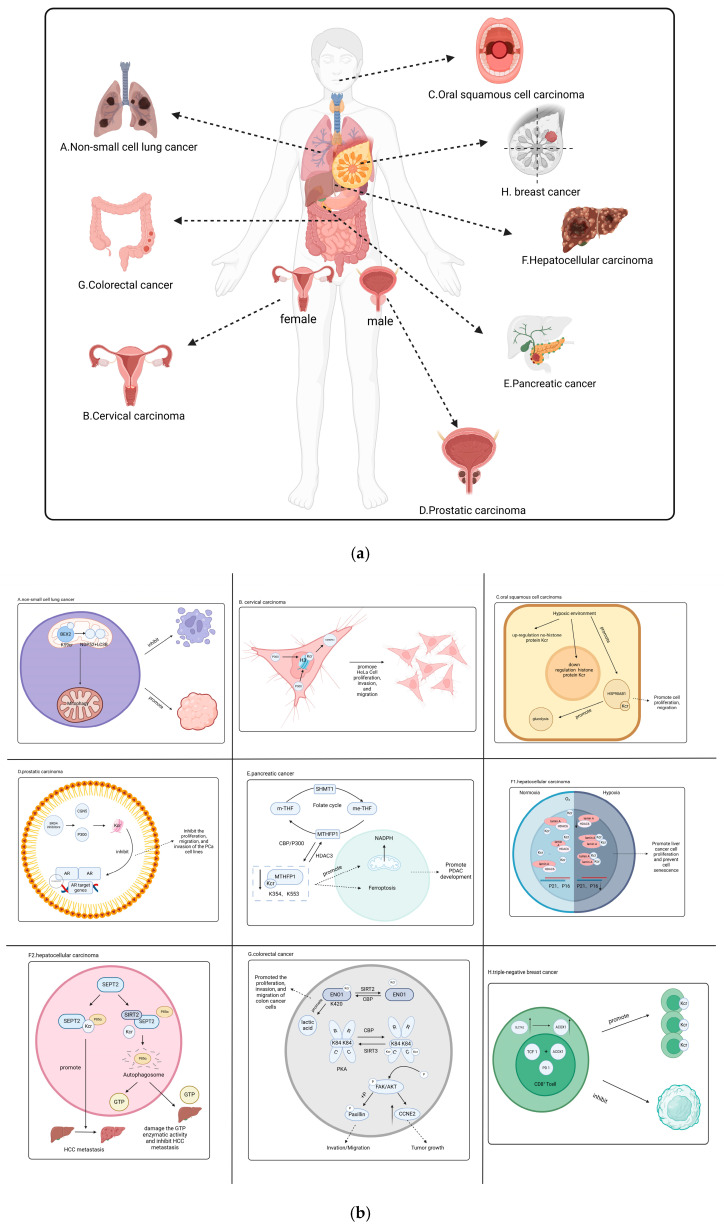
The regulatory mechanisms of crotonylation related to cancer. Figure 3a hows eight types of cancer associated with protein crotonylation. Figure 3b provides a detailed introduction to the crotonylation mechanism in eight cancer types. A is non-small cell lung cancer cell. B is cervical cancer cell. C is oral squamous cell carcinoma. D is prostate cancer. E is pancreatic cancer. F is liver cancer. G is colorectal cancer. H is triple-negative breast cancer. BEX2: human brain expressed X-linked 2; HNRNPA1: heterogeneous nuclear ribonucleoprotein A1; HSP90AB1: human heat shock protein 90 alpha family class B member 1; BRD4: bromodomain-containing protein 4; MTHFD1: methylenetetrahydrofolate dehydrogenase; HCC: hepatocellular carcinoma; ENO1: α enolase 1; PKA: protein kinase A; CRC: colorectal cancer; ACOX1: acyl-coenzyme A oxidase 1; SLC7A2: solute carrier family 7 member 2; TCF1: T-cell factor 1.

Cancer poses a significant public health challenge that remains a leading cause of death on a global scale [75]. A recent quantitative proteomics study has characterized the p300-regulated lysine crotonylation, revealing that p300-targeted Kcr substrates may be linked to cancer. This suggests that crotonylation could act as a carcinogenic factor, promoting tumor progression. The EDRN database indicates that 4.5% (20 out of 443) of tumor biomarkers have undergone crotonylation, and 32 crotonylated proteins are associated with tumor-related genes. These crotonylated proteins play a crucial role in tumorigenesis and development [76]. In adrenal aldosterone-producing adenomas, the crotonylated protein-associated gene is ATPase Na^+^/K^+^ transporting subunit alpha 1 (ATP1A1). In Spitzoid tumors, the relevant genes include lamin A/C (LMNA) and lamin B2 (LMNB2). In the case of anaplastic large cell lymphoma, the genes linked to protein crotonylation are moesin (MSN), myosin heavy chain 9 (MYH9), and myosin heavy chain 10 (MYH10). Further exploration is needed to understand the role of crotonylation in these proteins associated with tumor-related genes [25]. Various studies have found that crotonylation is downregulated in hepatic carcinoma, stomach cancer, and renal carcinoma while being upregulated in thyroid cancer, esophageal cancer, colorectal carcinoma, pancreatic cancer, and lung cancer, suggesting that crotonylation may play a role by regulating different cancer-related proteins [77]. In addition, protein crotonylation was reported in all eight cervical cancer subtypes [77]. Therefore, further study of the role of Kcr in tumors is conducive to developing new targeted drugs. This paper summarizes eight types of crotonylation-related cancers, and their regulatory mechanisms are shown in Figure 3.

### 3.1. Non-Small Cell Lung Cancer

Non-small-cell lung cancer (NSCLC) is the major subtype of lung cancer. Notably, an LC-MS/MS study revealed a number of crotonylated proteins in the H1299 NSCLC cell line [23]. Recently, it was shown that crotonylation of brain-expressed X-linked gene family (BEX2) at K59 promotes the interaction between NDP52 and LC3B, enhances mitochondrial autophagy, and promotes tumor growth in human lung cancer cells while also inhibiting chemotherapy-induced apoptosis [78]. Moreover, chemotherapeutic treatment resulted in increased BEX2 expression and increased cell viability. In addition, BEX2 was also localized in mitochondria. After H1299 cells were treated with the mitochondrial phagocytosis inducer CCCP, the localization of BEX2 in mitochondria was increased. In addition, BEX2 promoted the interaction between NDP52 and LC3B, thus enhancing the mitosis of NSCLC cells. Conversely, inhibition of BEX2-regulated mitochondrial autophagy increased the sensitivity of tumor cells to apoptosis. Therefore, mitochondrial autophagy induced by BEX2 crotonylation may be a promising target for NSCLC treatment.

### 3.2. Cervical Carcinoma

Cervical cancer is the third most common cancer in women and presents significant challenges in clinical treatment [79]. It has been shown that Kcr may affect the progression of cervical cancer [76]. One of the most important pathogenic factors is human papillomavirus (HPV), with well-known HPV-associated cervical cancer cell lines including HeLa, CaSki, and SiHa. Heterogeneous ribonucleic acid protein A1(HNRNPA1) is a potential cancer biomarker closely related to the occurrence and development of tumors and is involved in the apoptosis of colon cancer cells [79]. Its expression is closely related to the proliferation, invasion, and nuclear migration of HeLa cells. Studies have shown that knocking down HNRNPA1 can weaken cell proliferation, invasion, and migration. Knocking down P300 can lead to decreased Kcr levels in HeLa cells, and extra crotonyl-coA produced by NaCr can induce P300-mediated Kcr [34]. The level of Kcr can be saved by adding NaCr and can promote HNRNPA1 expression. Therefore, the downregulation of lysine crotonylation mediated by p300 can regulate the expression of HNRNPA1 by adding NaCr. Thus, enhancing lysine crotonylation enhances cell development and promotes the proliferation, invasion, and migration of HeLa cells [79]. Other crotonylation-regulated proteins, such as SIRT1 [33], SIRT2 [80], and SIRT3 [81], have all been confirmed to play a regulatory role in cervical cancer. However, the specific regulatory mechanism of crotonylation has not been confirmed, so more in-depth studies are needed.

### 3.3. Oral Squamous Cell Carcinoma

Oral squamous cell carcinoma (OSCC) is a common head and neck malignant tumor characterized by heterogeneity and aggressiveness [82]. Tumor hypoxia is considered an important prognostic factor, as it influences metabolic pathways, stimulates angiogenesis, and increases resistance to chemotherapy [83]. The role of anoxia in OSCC has been demonstrated in numerous studies [84,85,86]. Heat shock protein 90 is composed of heat shock protein 90α family class A member 1 (HSP90 AA1), heat shock protein 90α family class B member 1 (HSP90 AB1), glucose regulatory protein 94 (GRP 94), and TNF receptor-associated protein 1 (TRAP1). It acts as a molecular chaperone and is involved in the development and progression of cancer by stabilizing and activating client proteins involved in cell growth and survival. Studies have shown that increased expression of HSP90 AB1 in head and neck squamous cell carcinoma (HNSCC) is correlated with T grade, lymph node metastasis, and a poor prognosis, while the knockout of HSP90 AB1 was found to inhibit the proliferation, migration, and glycolytic activity of cultured HNSCC cells [87]. A recent study exploring oral squamous cell carcinoma under hypoxic conditions through subcellular localization found that upregulated crotonylated proteins were enriched in non-histones, and down-regulated crotonylated proteins were enriched in histones. After bioinformatics analysis, it was found that both upregulated and downregulated crotonylation proteins are related to binding and catalytic activity. Glycolysis-related proteins are prominent in upregulated proteins, with HSP90AB1 showing the most significant upregulation. It has been proved that HSP90AB1 is involved in regulating glycolysis in OSCC [87]. By mutating the K265 site of HSP90AB1 to prevent crotonylation and exploring the regulation ability in glycolysis, it was found that lactic acid levels and ATP in the mutant group were significantly lower than those of the wild type [62]. Glycolysis promotes tumor growth [88]. This demonstrates that crotonylation under hypoxic conditions may promote glycolysis, which may be conducive to treating OSCC [62].

### 3.4. Prostatic Carcinoma

Prostatic carcinoma (PCa) is the most common genitourinary malignancy in middle-aged and older men [89]. Studies have shown that Kcr plays a role in transcription, protein stability, regulation of metabolic pathways as well as the tumorigenesis and progression of PCa [7,90]. Bromodomain protein 4 (BRD4) is a member of the bromodomain and extra-terminal domain protein family and is also an effector protein of histone acetylation. BRD4 can bind to histone acetylation sites and interact with androgen receptor signaling proteins in tumor cells [91,92,93]. Recent studies found that BRD4 can regulate histone crotonylation in prostate cancer cells by regulating the expression levels of histone acetyltransferase, GCN5, and p300, while in androgen-dependent prostate cancer cell line LNCaP and castration-resistant prostate cancer cell line C42B, histone crotonylation can specifically activate the androgen receptor signaling pathway, thereby promoting cell proliferation, migration, and invasion [60]. Therefore, histone Kcr has great significance for the treatment of prostate cancer.

### 3.5. Pancreatic Cancer

Pancreatic cancer is a highly aggressive and deadly malignancy that is more common in men and the elderly. It accounts for 2% of all cancers but 5% of all cancer-related deaths [94]. A recent study demonstrated that most metabolic enzymes are crotonylated in pancreatic ductal adenocarcinoma (PDAC), including enzymes related to glycolysis, TCA cycle, fatty acid (FA) metabolism, glutamine metabolism, glutathione (GSH) metabolism, urea cycle, one-carbon metabolism, and mitochondrial fusion/fission kinetics [95]. Changes in metabolic enzymes are mainly dependent on the availability of intermediates, such as crotonyl-CoA, which are mainly regulated by crotonyltransferases (p300/CBP and MOF) and decrotonylases (HDAC1, HDAC2, HDAC3, and HDAC8) [95]. It has been demonstrated that pancreatic carcinogenesis can lead to high expression of histone deacetylases HDAC1 and HDAC2, resulting in downregulation of e-cadherin, thus promoting the epithelial–mesenchymal transition (EMT) and metastasis [96]. In addition, HDAC3-mediated deacetylation of ENO2 can lead to the activation of ENO2 and the enhancement of glycolysis. Through experiments, it has been proven that p300/CBP, HDAC1, and HDAC3 can lead to the crotonylation and de-crotonylation of MTHFD at K354 and K553. Low crotonylation can promote the generation of NADPH, inhibit iron apoptosis, and promote the development of PDAC tumors. The folate pathway enzyme methylenetetrahydrofolate dehydrogenase 1 is a cytoplasmic triple-functional enzyme providing a one-carbon tetrahydrofolate derivative. CBP/P300 and HDAC3 act as the crotonyltransferase and decarboxylase of MTHFD1, respectively, in PDAC. When MTHFD1 undergoes de-crotonylation at 354 and 553 sites, cells’ resistance to iron death increases, thus promoting the development of pancreatic cancer cells [95]. Therefore, studying crotonylation may provide new potential therapeutic targets for preventing and treating this deadly malignancy.

### 3.6. Hepatocellular Carcinoma

Hepatocellular carcinoma (HCC) is one of the most common and deadly types of cancer and the third leading cause of cancer-related deaths worldwide [97,98,99]; HCC mainly occurs in patients with underlying chronic liver disease and cirrhosis [98]. Kcr plays an essential role in metabolic homeostasis and liver cancer progression [59]. Wan et al. decreased the content of HDAC1 and HDAC3 using siRNA or the HDAC inhibitor TSA, which increased the total crotonylation level of cultured HCC cells. TSA-induced cancer cell cycle arrest, differentiation, and cell death through gene expression changes and alterations in histones and other proteins [100]. Another study found that such changes impede the movement and proliferation of liver cancer cells [77]. Zhang et al. demonstrated that crotonylation of proteins is very sensitive to fluctuations of oxygen concentration, whereby hypoxia promotes crotonylation, the interaction of HDAC6 with Lamin A contributes to crotonylation, and HDAC6 is downregulated under hypoxia conditions, which leads to the crotonylation of Lamin A and increased proliferation of HCC. Induction of p21 and p16 expression prevented crotonylation of Lamin A at K265 and K270, inhibiting senescence and proliferation of HCC cells [46]. After determining the global crotonylation levels of 100 HCC tissue samples, Kcr was positively correlated with the epithelial–mesenchymal transformation (EMT) [101]. Using NaCr to increase the total crotonylation levels promoted the EMT, resulting in increased migration and invasion of HCC cells. Recent studies revealed a new role of crotonylation in promoting liver cancer metastasis and invasion. Septin2 (SEPT2) is a member of the septin family of GTPases [102], which is essential for cytoplasmic division [103]. Crotonylation was found to be positively correlated with HCC cell migration and invasion. SEPT2 is the protein with the highest level of crotonylation in highly invasive cells, and previous studies have shown that SEPT2 can promote tumor growth and metastasis. SIRT2 can de-crotonate SEPT2. The K74 site of SEPT2 is located in the GTPase domain. A lysine mutation at the K74 site can significantly eliminate SEPT2’s crotonylation, inhibit cell growth and cell cycle, and suppress HCC metastasis. P85 α is a subunit of PI3K that is important for EMT. It interacts with SEPT2 to regulate mitosis. Its GTPase activity is crucial for mitosis. The study found that K74R mutation impairs the interaction between SEPT2 and P85 α, enhances the stability of P85 α by affecting its autophagy degradation, and thereby inhibits HCC metastasis in vitro and in vivo. Mechanistically, crotonylated SEPT2 enhanced invasion by upregulating the activity of the P85α-Akt pathway [101]. Hyper-crotonylation of SEPT2 at K74 predicts a high risk of recurrence in HCC patients [101]. A new article shows that P53 deficiency enhances the cytotoxic tolerance of human colon cancer cells to cisplatin treatment. In colon cancer cells with p53 deficiency, the significant increase in K283 crotonylation in cisplatin resistance-related genes (RRM2) leads to increased cisplatin resistance-related genes ATP7A, ATP7B, and MUC16, while the expression of cleaned-PARP1 and cleaned-caspase3 decreases. Therefore, the increase in RRM2 K283cr can enhance the cytotoxic tolerance of colon cancer cells to cisplatin treatment after p53 knockout, and SIRT7 was found to be its de-crotonylation enzyme. Therefore, p53 deficiency-induced cisplatin resistance can be overcome through the P53/SIRT7/RRM2 K283cr/cleaned-PARP1 and cleaned-caspase3 axes [104]. Protein crotonylation is closely related to HCC.

### 3.7. Colorectal Cancer

Colorectal cancer (CRC) is a leading cause of cancer-related deaths worldwide [105,106]. ENO1 overexpression in colon cancer tissues is associated with disease progression [107]. ENO1 overexpression was also found to promote the proliferation, migration, and invasion of tumor cells in vitro, and can promote the occurrence and metastasis of tumors in vivo [108]. Hou et al. demonstrated that the increase in ENO1 Kcr in colon cancer tissues is independent of ENO1 protein levels because CBP promotes the crotonylation of ENO1, and SIRT2 reduces its Kcr level. Functional analysis of the ENO1 crotonylation site showed that the K420 site is the leading Kcr site of ENO1. ENO1, as a glycolytic enzyme, is crucial for cancer cell energetics. The ENO1 K420cr mutant shows more vigorous ENO1 activity than wild-type ENO1 and can promote lactate production inside and outside rectal cancer cells. Lactate, a product of glycolysis, has also been found to be a key regulator of cancer development. High lactate helps cancer cells resist the stress of glucose deprivation, which often leads to apoptosis and/or necrosis. ENO1-k420 Kcr increased the activity of ENO1 and the content of lactic acid both inside and outside of CRC cells. Increased glycolysis in ENO1-K420cr cells raised the possibility that CRC cells may be insensitive to glucose depletion. Its carcinogenic effect is based on the increased viability of CRC cells due to increased lactic acid levels in the absence of glucose, which promotes proliferation, invasion, and migration [68]. This represents a promising new therapeutic target. Liao et al. reported that croconic acid (CA, 4,5-dihydroxycyclopentenetrione) produced by intestinal microbiota can enhance the proliferation of CRC cells by promoting the crotonylation of p53-Ser46 [22]. Liao et al. proposed that histone crotonylation may be involved in DNA damage. It has been demonstrated that Kcr of RPA1 protein mediates the repair of DNA damage induced by camptothecin (CPT) [109], and the crotonylation level of H3K9 decreased during the DNA damage period [110]. Studies confirmed that SIRT6 could reduce the crotonylation level of H3K27, and in colon cancer patients, the expression of genes whose promoters are occupied by histone 3 crotonylated at K27 was negatively correlated with DNA damage [52]. The gene LINC00922 encodes a long non-coding RNA (lncRNA) that can promote the proliferation, migration, and invasion of colon, lung, stomach, ovarian, and liver cancer cells. It has been shown that it can recruit DNA methyltransferases (DNMTs) to the promoter regions of target genes, thereby causing changes in their expression levels [111]. Liao et al. showed that LINC00922 is involved in the invasion and migration of CRC cells, interacts with SIRT3, and mediates its translocation to the ETS1 promoter, where it increases H3K27 crotonylation and increases expression, thus promoting the invasion and migration of CRC cells in vivo and vitro [112]. Hou et al. found that the Kcr of protein kinase A (PKA) holoenzyme in the cAMP signaling pathway center is upregulated. Nearly half of the proteins closely related to protein kinase A (PRKACA) are regulatory subunits of PKA, indicating that the Kcr of PRKACA may play a role in the interaction between PRKACA and regulatory subunits. The crotonylation of cAMP-dependent catalytic subunit α of PRKACA was increased in all three tested CRC cell lines (HCT116, SW480, and SW620). Crotonylation of PRKACA was reduced by siRNA knockdown of endogenous CBP or an increase in SIRT3 in HCT116 cells. Co-transfection offset the downregulation of crotonylation by SIRT3 and the upregulation of crotonylation by CBP. PKA is a tetramer consisting of two inactive catalytic subunits (C) and two regulatory subunits (R). The main crotonylation site of PRKACA is K84 and cells with increased crotonylation showed higher cAMP-dependent protein kinase (PKA) activity. It is speculated that crotonylation of K84 may reduce the binding of regulatory and catalytic subunits, thus activating PKA and leading to the phosphorylation of focal adhesion kinase (FAK) and protein kinase B (AKT) at the same time. The research found that the proliferation rates, colony growth, invasion, and migration of CRC cells with K84Q overexpression were significantly higher than those of wild-type cells while decreasing in K84R cells. In addition, K84Q elevated the mRNA levels of cyclin E2 (CCNE2), a classic gene associated with tumor cell growth. The phosphorylation levels of FAK at Y397, Akt at S473, Paxillin (a focus adhesion marker) at Y118, and the protein level of CCNE2 increased in K84Q cells, which PKA inhibitor H89 can reverse. These results indicate that PRKACA K84Q enhances PKA activity and phosphorylates FAK and Akt, resulting in downstream CCNE2 expression and Paxillin phosphorylation in a PKA-dependent way. Therefore, studies suggest that crotonylation at the K84 site of PRKACA can promote the growth, invasion, and migration of CRC cells by activating the PKA-FAK-AKT signaling pathway, indicating that it may be a diagnostic marker [113]. Liu et al. found that crotonylation of H3K27 resulted in transcriptional suppression and was selectively recognized by the YEATS domain of glioma-amplified sequence 41 (GAS41) and the SIN 3A-HDAC1 co-repressor complex. The proto-oncogene transcription factor MYC recruits the GAS 41/SIN 3A-HDAC1 complex to densify chromatin, including the cell cycle inhibitor. Knockout of GAS41 or crotonylation of H3K27 resulted in p21 downregulation, cell cycle arrest, and tumor growth inhibition in colon cancer [114]. Hou et al. analyzed crotonylation in colorectal cancer and para-cancer normal tissues, finding that some crotonylated proteins were enriched in immune-related pathways. Previous studies have shown that immune cells circulate between peripheral blood and tumor tissues [115,116], indicating the possibility of differential crotonylation in peripheral blood immune cells of patients with colorectal cancer. It was reported that the crotonylation level of H2BK12 in peripheral blood mononuclear cells of patients with rectal cancer was significantly increased, and the high level of H2BK12 crotonylation is closely related to advanced tumor stage, indicating that it may be a potential diagnostic marker for colorectal cancer [117].

### 3.8. Breast Cancer

Breast cancer is the most common malignancy in women worldwide, and triple-negative breast cancer (TNBC) is an aggressive subtype that accounts for 20% of all cases. Analysis of breast cancer cells under normoxic and hypoxic conditions showed that 128 Kcr sites were changed due to hypoxia, among which phosphoglycerate kinase 1 (PGK1) K131cr, K156cr, and K220cr were significantly reduced during hypoxia, PGK1 plays a crucial role in glycolysis by catalyzing the conversion of 1,3-diphosphoglycerate (1,3-BPG) and ADP to 3-phosphoglycerate (3-PG) and ATP, which is the first ATP-generating step. ECHS1 reduces PGK1 Kcr under hypoxia conditions, which will weaken mitochondrial pyruvate metabolism, promote the production of lactic acid, and promote tumor cell proliferation and migration. Studies have found that compared with early breast cancer cells, the level of PGK1 K131cr in advanced breast cancer cells is lower, which indicates that low levels of PGK1 K131cr are associated with poor prognosis in breast cancer patients. Therefore, PGK1 Kcr could be a promising candidate for diagnosing and treating breast cancer [118]. Solute carrier family 7 member 2 (SLC7A2) is a cationic amino acid transporter and a key regulator of lysine metabolism, as it transports cationic amino acids to the cytosol, leading to alterations in various biological processes such as cell proliferation and differentiation. It is often dysregulated in the pathogenesis of various cancers. Sun et al. found that removing SCL7A2 in CD8^+^ T cells can promote tumor cell proliferation and growth [61]. Moreover, SLC7A2 depletion decreases the ratios of CD8^+^ T cells and the infiltration of TNF-α+CD8^+^ T cells and IFN-γ+CD8^+^ T cells in vivo. These findings suggest that SLC7A2 enhances the tumor-killing ability of CD8^+^ T cells. SLC7A2-mediated lysine catabolism promotes the biogenesis of crotonyl-CoA and histone crotonylation (Kcr). They observed decreased mRNA expression of ACSS2, GCDH, ACADS, ACOX1, and ACOX3 and increased CDYL and ECHS1 expression. Moreover, ACOX1 expression was prominent. Overexpression of ACOX1 significantly antagonizes the depletion of SLC7A2 and inhibits tumor cell proliferation and growth. Exposure of cells to IFN-γ promotes the release of lys in CD8^+^ T cells, increases protein expression of SCL7A2, ACOX1, and Kcr, and inhibits cell viability and tumor growth. TCF1 is the critical transcription factor in CD8^+^ T cell regulation. ACOX1 and TCF1 co-localized in the nucleus of CD8^+^ T cells, overexpressed ACOX1 promoted the proliferation of total CD8^+^ T cells, TNF-α+CD8^+^ T cells, and IFN-γ+CD8^+^ T cells, as well as TCF1+PD-1+CD8^+^ T cells. Upregulation of SLC7A2 enhances ACOX1 expression and mediates CD8^+^ T cell histone Kcr to promote lysine release and inhibit tumor growth in TNBC patients. TCF1 plays critical roles in optimal priming of tumor antigen-specific CD8^+^ T cells, and TCF1+PD-1+CD8^+^ T lymphocytes mediate its proliferative response to ICB immunotherapies. Nuclear ACOX1 can interact with TCF1, promote the proliferation of TCF+PD-1+CD8^+^ stem cell-like T cells, improve the chemosensitivity of breast cancer cells to ICB, and inhibit the growth of TNBC cells. Therefore, the SLC7A2-ACOX 1-crotonylated TCF 1 pathway of CD8^+^ T-cells may be a novel way to treat TNBC [61].

## 4. Detection of Crotonylation and Development of Anticancer Drugs

Due to the complex biochemical reactions required for the metabolism of ester drugs in the body, there is a lack of specific crotonylation enzymes, which limits further research on crotonylation drugs. The biological effects of HDACs, KATs, and bromo-domain inhibitors are typically associated with acetylation, but these drugs also regulate crotonylation. However, there is still a need for extensive research on how to specifically target protein crotonylation modifications for drug design and disease treatment. To elucidate the role of different lysine acylations, Wei et al. [43] and Liu et al. [36] designed and generated p300 (I1935G) and CBP (I1432G) mutants with HAT deficiency but vigorous HCT activity, as well as HDAC1/3 AGG-VRPP mutants lacking HDCR but with intact HDAC activity. 2017 Ju and He [119] designed a new coding scheme to predict protein crotonylation sites through a database. 2018, Bos and Muri [120] designed a chemical probe for endogenous protein crotonylation to supplement specific antibodies to detect crotonylation modifications. In 2019, Maleberry et al. [121] designed and developed a new database of protein crotonylation sites based on protein sequences’ physicochemical properties and evolutionary-derived features. In 2020, Spinck et al. [122] designed an HDAC-HDCR selection system in yeast by extending the genetic code and replacing an essential active site lysine residue of orotidine 5′-phosphate decarboxylase with a lysine derivative. In 2021, Xie et al. [123] developed single-step fluorescent probes (KTcr-I recognized by Sirt2 and KTcr-II recognized by HDAC3) that generate fluorescent signals through intramolecular nucleophilic exchange reactions to detect the de-crotonylation activity of HDACs. Lv et al. [124] used a deep learning-based method to accurately detect lysine crotonylation sites by combining sequence-based features, physicochemical property-based features, and numerical spatial derived information. Some HDAC families that inhibit crotonylation have been used for cancer treatment. Several HDAC inhibitors have been applied to treat T-cell lymphoma, Belinostat, and Panobinostat [125]. In addition, specific inhibitors targeting the “reader” protein of crotonylation have been applied clinically. For example, XL-13m is a selective inhibitor of the YEATS (YAF9, ENL, AF9, TAF14, SAS5) domain of ENL (Eleven Nineteen Leukemia), which can disrupt chromatin binding of ENL in vivo. This further reduces the expression of oncogenes in acute leukemia, with MLL (mixed lineage leukemia) gene rearrangement, and plays a crucial role in this disease [126].

## 5. Summary and Prospect

This review discussed and summarized the latest advances in Kcr research, including the regulatory mechanism of crotonylation, physiological functions, and its relationship with cancer. Crotonylation of lysine residues is a recently discovered evolutionarily conserved histone PTM [7]. Due to the advancement of high-resolution LC-MS/MS technology, protein crotonylation can be better studied. The identification of crotonylation in non-histones can also expand our understanding of other proteins involved in the post-translational regulation of different cellular functions and signaling pathways and could facilitate research into the precise regulation of protein function.

In recent years, the functional mechanisms of protein crotonylation of protein have been studied in increasing detail, but there are still many unknowns. Although Kcr is involved in various cellular functions in both health and disease states, there is no clear understanding of the specific mechanisms of action of Kcr in these biological processes. Changes in the balance between crotonylation and de-crotonylation have been implicated in a number of diseases, so a critical future research direction may be to gain insights into the target substrates of crotonylation and the role of this protein modification in biology. The concentration of crotonyl-CoA affects the crotonylation of both histones and other proteins [27]. Crotonyl-CoA is an intermediate in different metabolic processes, including the β-oxidation of fatty acids and degradation of the essential amino acids’ lysine or tryptophan, so it is also very important to study the factors influencing the intracellular pool of crotonyl-CoA. At present, the number of known writers, readers, and erasers of crotonylated lysine residues is very limited, and more proteins related to crotonylation can be discovered in future studies. More attention should also be paid to the regulatory mechanism of crotonylation in human health and disease. The role of crotonylation in non-histone proteins should be further explored, and more lysine crotonylation sites are expected to be discovered. There are many studies on the relationship between crotonylation and various cancers, but the actual underlying mechanism has not been discovered, and further research is needed. Since crotonylation overlaps with other acylation modifications, these epigenetic modifications are linked and work together to regulate gene expression. However, due to this overlap, there are significant obstacles to the specific study of crotonylation.

Moreover, the specific regulators and targets of Kcr have not been identified at the time of writing, which brings great challenges to research on crotonylation-mediated epigenetics and related fields. Therefore, it is particularly important to explore the specific mechanisms, regulators, and targets of Kcr in various diseases. In order to study crotonylation more profoundly and comprehensively, especially the roles and regulatory mechanisms of crotonylation in various cancers, it is necessary to conduct comprehensive research on the regulatory sites of crotonylation and design clinical trials to provide a fresh theoretical basis for clinical treatment.

## Figures and Tables

**Table 1:** Chemical structures and years of discovery of post-translational lysine modifications. According to their chemical properties, they are divided into three categories: hydrophobic acyl, acidic acyl, and polar acyl.

Classification	Histone Modification	Chemical Structure	Year	Ref
Hydrophobic acyl group	Acetylation (Kac)	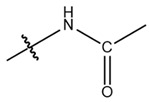	1962	[4]
	Propionylation (Kpr)	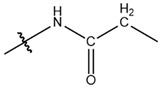	2007	[5]
	Butyrylation (Kbu)	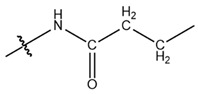	2007	[6]
	Crotonylation (Kcr)	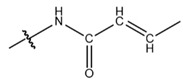	2011	[7]
	Benzoylation (Kbz)	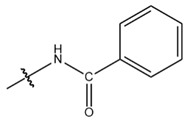	2018	[8]
Acidic acyl group	Malonylation (Kmal)	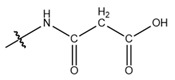	2012	[10]
	Succinylation (Ksucc)	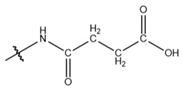	2012	[11]
	Glutarylation (Kglu)	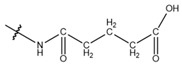	2014	[12]
Polar acyl group	2-hydroxyisobutyrylation (Khib)	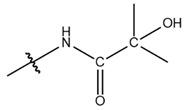	2014	[13]
	β-hydroxybutyrylation (Kbhb)	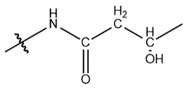	2016	[14]
	Lactylation (Kla)	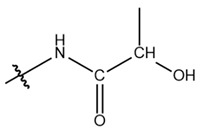	2019	[15]

**Table 2:** Regulatory factors of crotonylation, their classification, and years of discovery.

Regulation Patter	Family	Members	Histone Crotonylation Site	Non-Histone Target Proteins	Year	Ref
Writers	P300/CBP	P300	H3K18		2015	[34]
		CBP	H3K18	NPM1, DDX5, ENO1	2015, 2017, 2001	[34] [23] [35]
	MYST	MOF	H3K4, H3K9, H3K18, H3K23, H4K8, H4K12		2017, 2017	[36] [23]
		ESa1 (Piccolo NuA4)	H4K5, H4K8, H4K12, H4K16		2019	[26]
	GCN5 (ADA)		H3K9, H3K14, H3K18, H3K23, H3K27		2019	[26]
		TIP60		EB1	2021	[37]
		KAT7		CANX (K525)	2022	[38]
		PCAF		NPM1, DDX5	2017	[23]
Readers	Bromodomain family	TAF1			2016, 2015	[39] [40]
	YEATS	TAF14	H3K9		2016	[41]
		YEATS2	H3K27		2016	[42]
		AF9	H3K9, H3K18, H3K27		2016	[39]
	DPF	MOZ	H3K14		2016	[24]
		DPF2	H3K14		2016	[24]
Erasers	Class Ⅰ	HDAC1	H3K4, H3K9, H3K18, H3K23, H4K8, H4K12		2017, 2023, 2017, 2023	[23] [33] [43] [44]
		HDAC2	H3K9, H3K18, H3K23, H4K8, H4K12, H2BK12		2021, 2017	[45] [43]
		HDAC3	H3K9, H3K18, H4K8	NPM1, AKT1	2017, 2023, 2017, 2023	[23] [33] [43] [44]
		HDAC8	H3K4, H3K9, H3K18, H3K23, H4K8, H4K12		2021, 2017	[45] [43]
	Class Ⅱ	HDAC6		Lamin A (K265/270)	2022	[46]
		HDAC7		14-3-3ϵ (K73, K78)	2022	[47]
		HDAC5 (Fosir5)	H3K18		2021	[48]
	Class Ⅲ	SIRT1	H3K4, H3K9, H3K18, H4K8, H2AK119		2017, 2013, 2022	[43] [49] [50]
		SIRT2	H3K9	ENO1	2001, 2013	[35] [49]
		SIRT3	H3K4		2014	[51]
		SIRT6	H3K27		2022	[52]
		SIRT7		PHF5A (K25)	2021	[53]
Others		CDYL	H3K9, H3K18, H3K23, H3K27, H3K79, H4K8, H2BK12		2017, 2017	[54] [55]
		ESHS1	H3K18, H2BK12		2021	[56]
		GCDH			2000	[57]
		ACSS2	H3K4, H3K18		2015, 2018	[34] [58]
		ACOX2	H2BK86	EHHADH (K329R, K344R) CROT (K69R, K384R)	2022	[59]
		ACOX3			2021	[28]
		ACADS			2021	[28]
		BRD4	H3K18		2021	[60]
		ACOX1			2024	[61]
		Rate-limiting enzyme			2023	[62]

## References

[1] Jiang G., Li C., Lu M., Lu K., Li H. (2021). Protein lysine crotonylation: Past, present, perspective. Cell Death Dis..

[2] Wang R., Wang G. (2019). Protein Modification and Autophagy Activation. Adv. Exp. Med. Biol..

[3] Seo J., Lee K.J. (2004). Post-translational modifications and their biological functions: Proteomic analysis and systematic approaches. J. Biochem. Mol. Biol..

[4] Sutendra G., Kinnaird A., Dromparis P., Paulin R., Stenson T.H., Haromy A., Hashimoto K., Zhang N., Flaim E., Michelakis E.D. (2014). A nuclear pyruvate dehydrogenase complex is important for the generation of acetyl-CoA and histone acetylation. Cell.

[5] Chen Y., Sprung R., Tang Y., Ball H., Sangras B., Kim S.C., Falck J.R., Peng J., Gu W., Zhao Y. (2007). Lysine propionylation and butyrylation are novel post-translational modifications in histones. Mol. Cell Proteom..

[6] Van Slyke D.D., Sinex F.M. (1958). The course of hydroxylation of lysine to form hydroxylysine in collagen. J. Biol. Chem..

[7] Tan M., Luo H., Lee S., Jin F., Yang J.S., Montellier E., Buchou T., Cheng Z., Rousseaux S., Rajagopal N. (2011). Identification of 67 histone marks and histone lysine crotonylation as a new type of histone modification. Cell.

[8] Huang H., Zhang D., Wang Y., Perez-Neut M., Han Z., Zheng Y.G., Hao Q., Zhao Y. (2018). Lysine benzoylation is a histone mark regulated by SIRT2. Nat. Commun..

[9] Barnes C.E., English D.M., Cowley S.M. (2019). Acetylation & Co: An expanding repertoire of histone acylations regulates chromatin and transcription. Essays Biochem..

[10] Xie Z., Dai J., Dai L., Tan M., Cheng Z., Wu Y., Boeke J.D., Zhao Y. (2012). Lysine succinylation and lysine malonylation in histones. Mol. Cell. Proteom..

[11] Zhang Z., Tan M., Xie Z., Dai L., Chen Y., Zhao Y. (2011). Identification of lysine succinylation as a new post-translational modification. Nat. Chem. Biol..

[12] Tan M., Peng C., Anderson K.A., Chhoy P., Xie Z., Dai L., Park J., Chen Y., Huang H., Zhang Y. (2014). Lysine glutarylation is a protein posttranslational modification regulated by SIRT5. Cell Metab..

[13] Dai L., Peng C., Montellier E., Lu Z., Chen Y., Ishii H., Debernardi A., Buchou T., Rousseaux S., Jin F. (2014). Lysine 2-hydroxyisobutyrylation is a widely distributed active histone mark. Nat. Chem. Biol..

[14] Xie Z., Zhang D., Chung D., Tang Z., Huang H., Dai L., Qi S., Li J., Colak G., Chen Y. (2016). Metabolic Regulation of Gene Expression by Histone Lysine β-Hydroxybutyrylation. Mol. Cell.

[15] Zhang D., Tang Z., Huang H., Zhou G., Cui C., Weng Y., Liu W., Kim S., Lee S., Perez-Neut M. (2019). Metabolic regulation of gene expression by histone lactylation. Nature.

[16] Figlia G., Willnow P., Teleman A.A. (2020). Metabolites Regulate Cell Signaling and Growth via Covalent Modification of Proteins. Dev Cell.

[17] Sun H., Liu X., Li F., Li W., Zhang J., Xiao Z., Shen L., Li Y., Wang F., Yang J. (2017). First comprehensive proteome analysis of lysine crotonylation in seedling leaves of Nicotiana tabacum. Sci. Rep..

[18] Kwon O.K., Kim S.J., Lee S. (2018). First profiling of lysine crotonylation of myofilament proteins and ribosomal proteins in zebrafish embryos. Sci. Rep..

[19] Yang Q., Li Y., Apaliya M.T., Zheng X., Serwah B.N.A., Zhang X., Zhang H. (2018). The Response of Rhodotorula mucilaginosa to Patulin Based on Lysine Crotonylation. Front. Microbiol..

[20] Zhu J., Dong Q., Dong C., Zhang X., Zhang H., Chen Z. (2020). Global Lysine Crotonylation Alterations of Host Cell Proteins Caused by Brucella Effector BspF. Front. Cell. Infect. Microbiol..

[21] Lin R., Li A., Li Y., Shen R., Du F., Zheng M., Zhu J., Chen J., Jiang P., Zhang H. (2023). The Brucella Effector Protein BspF Regulates Apoptosis through the Crotonylation of p53. Microorganisms.

[22] Liao P., Bhattarai N., Cao B., Zhou X., Jung J.H., Damera K., Fuselier T.T., Thareja S., Wimley W.C., Wang B. (2020). Crotonylation at serine 46 impairs p53 activity. Biochem. Biophys. Res. Commun..

[23] Xu W., Wan J., Zhan J., Li X., He H., Shi Z., Zhang H. (2017). Global profiling of crotonylation on non-histone proteins. Cell Res..

[24] Xiong X., Panchenko T., Yang S., Zhao S., Yan P., Zhang W., Xie W., Li Y., Zhao Y., Allis C.D. (2016). Selective recognition of histone crotonylation by double PHD fingers of MOZ and DPF2. Nat. Chem. Biol..

[25] Wang S., Mu G., Qiu B., Wang M., Yu Z., Wang W., Wang J., Yang Y. (2021). The Function and related Diseases of Protein Crotonylation. Int. J. Biol. Sci..

[26] Martinez-Moreno J.M., Fontecha-Barriuso M., Martín-Sánchez D., Sánchez-Niño M.D., Ruiz-Ortega M., Sanz A.B., Ortiz A. (2020). The Contribution of Histone Crotonylation to Tissue Health and Disease: Focus on Kidney Health. Front. Pharmacol..

[27] Li K., Wang Z. (2021). Histone crotonylation-centric gene regulation. Epigenetics Chromatin.

[28] Fang Y., Xu X., Ding J., Yang L., Doan M.T., Karmaus P.W.F., Snyder N.W., Zhao Y., Li J.L., Li X. (2021). Histone crotonylation promotes mesoendodermal commitment of human embryonic stem cells. Cell Stem Cell.

[29] Biagosch C., Ediga R.D., Hensler S.V., Faerberboeck M., Kuehn R., Wurst W., Meitinger T., Kölker S., Sauer S., Prokisch H. (2017). Elevated glutaric acid levels in Dhtkd1-/Gcdh- double knockout mice challenge our current understanding of lysine metabolism. Biochim. Biophys. Acta Mol. Basis Dis..

[30] Fischer C.R., Tseng H.C., Tai M., Prather K.L., Stephanopoulos G. (2010). Assessment of heterologous butyrate and butanol pathway activity by measurement of intracellular pathway intermediates in recombinant Escherichia coli. Appl. Microbiol. Biotechnol..

[31] Li D., Lin L., Xu F., Feng T., Tao Y., Miao H., Yang F. (2024). Protein crotonylation: Basic research and clinical diseases. Biochem. Biophys. Rep..

[32] Li W., Long Q., Wu H., Zhou Y., Duan L., Yuan H., Ding Y., Huang Y., Wu Y., Huang J. (2022). Nuclear localization of mitochondrial TCA cycle enzymes modulates pluripotency via histone acetylation. Nat. Commun..

[33] Yang P., Qin Y., Zeng L., He Y., Xie Y., Cheng X., Huang W., Cao L. (2023). Crotonylation and disease: Current progress and future perspectives. Biomed. Pharmacother..

[34] Sabari B.R., Tang Z., Huang H., Yong-Gonzalez V., Molina H., Kong H.E., Dai L., Shimada M., Cross J.R., Zhao Y. (2015). Intracellular crotonyl-CoA stimulates transcription through p300-catalyzed histone crotonylation. Mol. Cell.

[35] Jenuwein T., Allis C.D. (2001). Translating the histone code. Science.

[36] Liu X., Wei W., Liu Y., Yang X., Wu J., Zhang Y., Zhang Q., Shi T., Du J.X., Zhao Y. (2017). MOF as an evolutionarily conserved histone crotonyltransferase and transcriptional activation by histone acetyltransferase-deficient and crotonyltransferase-competent CBP/p300. Cell Discov..

[37] Song X., Yang F., Liu X., Xia P., Yin W., Wang Z., Wang Y., Yuan X., Dou Z., Jiang K. (2021). Dynamic crotonylation of EB1 by TIP60 ensures accurate spindle positioning in mitosis. Nat. Chem. Biol..

[38] Yan G., Li X., Zheng Z., Gao W., Chen C., Wang X., Cheng Z., Yu J., Zou G., Farooq M.Z. (2022). KAT7-mediated CANX (calnexin) crotonylation regulates leucine-stimulated MTORC1 activity. Autophagy.

[39] Li Y., Sabari B.R., Panchenko T., Wen H., Zhao D., Guan H., Wan L., Huang H., Tang Z., Zhao Y. (2016). Molecular Coupling of Histone Crotonylation and Active Transcription by AF9 YEATS Domain. Mol. Cell.

[40] Flynn E.M., Huang O.W., Poy F., Oppikofer M., Bellon S.F., Tang Y., Cochran A.G. (2015). A Subset of Human Bromodomains Recognizes Butyryllysine and Crotonyllysine Histone Peptide Modifications. Structure.

[41] Andrews F.H., Shinsky S.A., Shanle E.K., Bridgers J.B., Gest A., Tsun I.K., Krajewski K., Shi X., Strahl B.D., Kutateladze T.G. (2016). The Taf14 YEATS domain is a reader of histone crotonylation. Nat. Chem. Biol..

[42] Zhao D., Guan H., Zhao S., Mi W., Wen H., Li Y., Zhao Y., Allis C.D., Shi X., Li H. (2016). YEATS2 is a selective histone crotonylation reader. Cell Res..

[43] Wei W., Liu X., Chen J., Gao S., Lu L., Zhang H., Ding G., Wang Z., Chen Z., Shi T. (2017). Class I histone deacetylases are major histone decrotonylases: Evidence for critical and broad function of histone crotonylation in transcription. Cell Res..

[44] Qian Z., Ye J., Li J., Che Y., Yu W., Xu P., Lin J., Ye F., Xu X., Su Z. (2023). Decrotonylation of AKT1 promotes AKT1 phosphorylation and activation during myogenic differentiation. J. Adv. Res..

[45] Yang J., He Z., Chen C., Li S., Qian J., Zhao J., Fang R. (2021). Toxoplasma gondii Infection Inhibits Histone Crotonylation to Regulate Immune Response of Porcine Alveolar Macrophages. Front. Immunol..

[46] Zhang D., Tang J., Xu Y., Huang X., Wang Y., Jin X., Wu G., Liu P. (2022). Global crotonylome reveals hypoxia-mediated lamin A crotonylation regulated by HDAC6 in liver cancer. Cell Death Dis..

[47] Zheng Z., Yan G., Li X., Fei Y., Sun L., Yu H., Niu Y., Gao W., Zhong Q., Yan X. (2022). Lysine crotonylation regulates leucine-deprivation-induced autophagy by a 14-3-3ε-PPM1B axis. Cell Rep..

[48] Zhang N., Song L., Xu Y., Pei X., Luisi B.F., Liang W. (2021). The decrotonylase FoSir5 facilitates mitochondrial metabolic state switching in conidial germination of Fusarium oxysporum. Elife.

[49] Feldman J.L., Baeza J., Denu J.M. (2013). Activation of the protein deacetylase SIRT6 by long-chain fatty acids and widespread deacylation by mammalian sirtuins. J. Biol. Chem..

[50] Hao S., Wang Y., Zhao Y., Gao W., Cui W., Li Y., Cui J., Liu Y., Lin L., Xu X. (2022). Dynamic switching of crotonylation to ubiquitination of H2A at lysine 119 attenuates transcription-replication conflicts caused by replication stress. Nucleic Acids Res..

[51] Bao X., Wang Y., Li X., Li X.M., Liu Z., Yang T., Wong C.F., Zhang J., Hao Q., Li X.D. (2014). Identification of ‘erasers’ for lysine crotonylated histone marks using a chemical proteomics approach. Elife.

[52] Liao M., Chu W., Sun X., Zheng W., Gao S., Li D., Pei D. (2022). Reduction of H3K27cr Modification During DNA Damage in Colon Cancer. Front. Oncol..

[53] Yu A.Q., Wang J., Jiang S.T., Yuan L.Q., Ma H.Y., Hu Y.M., Han X.M., Tan L.M., Wang Z.X. (2021). SIRT7-Induced PHF5A Decrotonylation Regulates Aging Progress Through Alternative Splicing-Mediated Downregulation of CDK2. Front. Cell Dev. Biol..

[54] Liu Y., Liu S., Yuan S., Yu H., Zhang Y., Yang X., Xie G., Chen Z., Li W., Xu B. (2017). Chromodomain protein CDYL is required for transmission/restoration of repressive histone marks. J. Mol. Cell Biol..

[55] Liu S., Yu H., Liu Y., Liu X., Zhang Y., Bu C., Yuan S., Chen Z., Xie G., Li W. (2017). Chromodomain Protein CDYL Acts as a Crotonyl-CoA Hydratase to Regulate Histone Crotonylation and Spermatogenesis. Mol. Cell.

[56] Tang X., Chen X.F., Sun X., Xu P., Zhao X., Tong Y., Wang X.M., Yang K., Zhu Y.T., Hao D.L. (2021). Short-Chain Enoyl-CoA Hydratase Mediates Histone Crotonylation and Contributes to Cardiac Homeostasis. Circulation.

[57] Dwyer T.M., Rao K.S., Goodman S.I., Frerman F.E. (2000). Proton abstraction reaction, steady-state kinetics, and oxidation-reduction potential of human glutaryl-CoA dehydrogenase. Biochemistry.

[58] Jiang G., Nguyen D., Archin N.M., Yukl S.A., Méndez-Lagares G., Tang Y., Elsheikh M.M., Thompson G.R., Hartigan-O’Connor D.J., Margolis D.M. (2018). HIV latency is reversed by ACSS2-driven histone crotonylation. J. Clin. Invest..

[59] Bruix J., Gores G.J., Mazzaferro V. (2014). Hepatocellular carcinoma: Clinical frontiers and perspectives. Gut.

[60] Xu X., Zhu X., Liu F., Lu W., Wang Y., Yu J. (2021). The effects of histone crotonylation and bromodomain protein 4 on prostate cancer cell lines. Transl. Androl. Urol..

[61] Sun Y., Li Y., Jiang C., Liu C., Song Y. (2024). SLC7A2-Mediated Lysine Catabolism Inhibits Immunosuppression in Triple Negative Breast Cancer. Crit. Rev. Eukaryot. Gene Expr..

[62] Yin X., Zhang H., Wei Z., Wang Y., Han S., Zhou M., Xu W., Han W. (2023). Large-Scale Identification of Lysine Crotonylation Reveals Its Potential Role in Oral Squamous Cell Carcinoma. Cancer Manag. Res..

[63] Hundertmark T., Gärtner S.M.K., Rathke C., Renkawitz-Pohl R. (2018). Nejire/dCBP-mediated histone H3 acetylation during spermatogenesis is essential for male fertility in Drosophila melanogaster. PLoS ONE.

[64] Kaczmarska Z., Ortega E., Goudarzi A., Huang H., Kim S., Márquez J.A., Zhao Y., Khochbin S., Panne D. (2017). Structure of p300 in complex with acyl-CoA variants. Nat. Chem. Biol..

[65] Ntorla A., Burgoyne J.R. (2021). The Regulation and Function of Histone Crotonylation. Front. Cell Dev. Biol..

[66] Decker P.V., Yu D.Y., Iizuka M., Qiu Q., Smith M.M. (2008). Catalytic-site mutations in the MYST family histone Acetyltransferase Esa1. Genetics.

[67] Kollenstart L., de Groot A.J.L., Janssen G.M.C., Cheng X., Vreeken K., Martino F., Côté J., van Veelen P.A., van Attikum H. (2019). Gcn5 and Esa1 function as histone crotonyltransferases to regulate crotonylation-dependent transcription. J. Biol. Chem..

[68] Hou J.Y., Cao J., Gao L.J., Zhang F.P., Shen J., Zhou L., Shi J.Y., Feng Y.L., Yan Z., Wang D.P. (2021). Upregulation of α enolase (ENO1) crotonylation in colorectal cancer and its promoting effect on cancer cell metastasis. Biochem. Biophys. Res. Commun..

[69] Klein B.J., Jang S.M., Lachance C., Mi W., Lyu J., Sakuraba S., Krajewski K., Wang W.W., Sidoli S., Liu J. (2019). Histone H3K23-specific acetylation by MORF is coupled to H3K14 acylation. Nat. Commun..

[70] Madsen A.S., Olsen C.A. (2012). Profiling of substrates for zinc-dependent lysine deacylase enzymes: HDAC3 exhibits decrotonylase activity in vitro. Angew. Chem. Int. Ed. Engl..

[71] Fellows R., Denizot J., Stellato C., Cuomo A., Jain P., Stoyanova E., Balázsi S., Hajnády Z., Liebert A., Kazakevych J. (2018). Microbiota derived short chain fatty acids promote histone crotonylation in the colon through histone deacetylases. Nat. Commun..

[72] Kelly R.D.W., Chandru A., Watson P.J., Song Y., Blades M., Robertson N.S., Jamieson A.G., Schwabe J.W.R., Cowley S.M. (2018). Histone deacetylase (HDAC) 1 and 2 complexes regulate both histone acetylation and crotonylation in vivo. Sci. Rep..

[73] Wei W., Mao A., Tang B., Zeng Q., Gao S., Liu X., Lu L., Li W., Du J.X., Li J. (2017). Large-Scale Identification of Protein Crotonylation Reveals Its Role in Multiple Cellular Functions. J. Proteome Res..

[74] Wang Z., Zhao Y., Xu N., Zhang S., Wang S., Mao Y., Zhu Y., Li B., Jiang Y., Tan Y. (2019). NEAT1 regulates neuroglial cell mediating Aβ clearance via the epigenetic regulation of endocytosis-related genes expression. Cell. Mol. Life Sci..

[75] Siegel R.L., Miller K.D., Jemal A. (2019). Cancer statistics, 2019. CA Cancer J. Clin..

[76] Huang H., Wang D.L., Zhao Y. (2018). Quantitative Crotonylome Analysis Expands the Roles of p300 in the Regulation of Lysine Crotonylation Pathway. Proteomics.

[77] Wan J., Liu H., Ming L. (2019). Lysine crotonylation is involved in hepatocellular carcinoma progression. Biomed. Pharmacother..

[78] Mu N., Wang Y., Li X., Du Z., Wu Y., Su M., Wang Y., Sun X., Su L., Liu X. (2023). Crotonylated BEX2 interacts with NDP52 and enhances mitophagy to modulate chemotherapeutic agent-induced apoptosis in non-small-cell lung cancer cells. Cell Death Dis..

[79] Han X., Xiang X., Yang H., Zhang H., Liang S., Wei J., Yu J. (2020). p300-Catalyzed Lysine Crotonylation Promotes the Proliferation, Invasion, and Migration of HeLa Cells via Heterogeneous Nuclear Ribonucleoprotein A1. Anal. Cell. Pathol..

[80] Kuhlmann N., Chollet C., Baldus L., Neundorf I., Lammers M. (2017). Development of Substrate-Derived Sirtuin Inhibitors with Potential Anticancer Activity. ChemMedChem.

[81] Xu L.X., Hao L.J., Ma J.Q., Liu J.K., Hasim A. (2020). SIRT3 promotes the invasion and metastasis of cervical cancer cells by regulating fatty acid synthase. Mol. Cell. Biochem..

[82] Solomon B., Young R.J., Rischin D. (2018). Head and neck squamous cell carcinoma: Genomics and emerging biomarkers for immunomodulatory cancer treatments. Semin. Cancer Biol..

[83] Bhandari V., Hoey C., Liu L.Y., Lalonde E., Ray J., Livingstone J., Lesurf R., Shiah Y.J., Vujcic T., Huang X. (2019). Molecular landmarks of tumor hypoxia across cancer types. Nat. Genet..

[84] Wei Z., Yin X., Cai Y., Xu W., Song C., Wang Y., Zhang J., Kang A., Wang Z., Han W. (2018). Antitumor effect of a Pt-loaded nanocomposite based on graphene quantum dots combats hypoxia-induced chemoresistance of oral squamous cell carcinoma. Int. J. Nanomed..

[85] Yin X., Han S., Song C., Zou H., Wei Z., Xu W., Ran J., Tang C., Wang Y., Cai Y. (2019). Metformin enhances gefitinib efficacy by interfering with interactions between tumor-associated macrophages and head and neck squamous cell carcinoma cells. Cell. Oncol..

[86] Yin X., Wei Z., Song C., Tang C., Xu W., Wang Y., Xie J., Lin Z., Han W. (2018). Metformin sensitizes hypoxia-induced gefitinib treatment resistance of HNSCC via cell cycle regulation and EMT reversal. Cancer Manag. Res..

[87] Zhang H., Yin X., Zhang X., Zhou M., Xu W., Wei Z., Song C., Han S., Han W. (2022). HSP90AB1 Promotes the Proliferation, Migration, and Glycolysis of Head and Neck Squamous Cell Carcinoma. Technol. Cancer Res. Treat..

[88] Martínez-Reyes I., Chandel N.S. (2021). Cancer metabolism: Looking forward. Nat. Rev. Cancer.

[89] Greiman A.K., Rosoff J.S., Prasad S.M. (2017). Association of Human Development Index with global bladder, kidney, prostate and testis cancer incidence and mortality. BJU Int..

[90] Zhao S., Zhang X., Li H. (2018). Beyond histone acetylation-writing and erasing histone acylations. Curr. Opin. Struct. Biol..

[91] Asangani I.A., Dommeti V.L., Wang X., Malik R., Cieslik M., Yang R., Escara-Wilke J., Wilder-Romans K., Dhanireddy S., Engelke C. (2014). Therapeutic targeting of BET bromodomain proteins in castration-resistant prostate cancer. Nature.

[92] Shi J., Vakoc C.R. (2014). The mechanisms behind the therapeutic activity of BET bromodomain inhibition. Mol. Cell.

[93] Kanno T., Kanno Y., LeRoy G., Campos E., Sun H.W., Brooks S.R., Vahedi G., Heightman T.D., Garcia B.A., Reinberg D. (2014). BRD4 assists elongation of both coding and enhancer RNAs by interacting with acetylated histones. Nat. Struct. Mol. Biol..

[94] Goral V. (2015). Pancreatic Cancer: Pathogenesis and Diagnosis. Asian Pac. J. Cancer Prev..

[95] Zheng Y., Zhu L., Qin Z.Y., Guo Y., Wang S., Xue M., Shen K.Y., Hu B.Y., Wang X.F., Wang C.Q. (2023). Modulation of cellular metabolism by protein crotonylation regulates pancreatic cancer progression. Cell Rep..

[96] Aghdassi A., Sendler M., Guenther A., Mayerle J., Behn C.O., Heidecke C.D., Friess H., Büchler M., Evert M., Lerch M.M. (2012). Recruitment of histone deacetylases HDAC1 and HDAC2 by the transcriptional repressor ZEB1 downregulates E-cadherin expression in pancreatic cancer. Gut.

[97] Yang J.D., Roberts L.R. (2010). Hepatocellular carcinoma: A global view. Nat. Rev. Gastroenterol. Hepatol..

[98] Siegel R.L., Miller K.D., Jemal A. (2018). Cancer statistics, 2018. CA Cancer J. Clin..

[99] Zhang Y., Chen Y., Zhang Z., Tao X., Xu S., Zhang X., Zurashvili T., Lu Z., Bayascas J.R., Jin L. (2022). Acox2 is a regulator of lysine crotonylation that mediates hepatic metabolic homeostasis in mice. Cell Death Dis..

[100] Hrabeta J., Stiborova M., Adam V., Kizek R., Eckschlager T. (2014). Histone deacetylase inhibitors in cancer therapy. A review. Biomed. Pap. Med. Fac. Univ. Palacky. Olomouc. Czech Repub..

[101] Zhang X.Y., Liu Z.X., Zhang Y.F., Xu L.X., Chen M.K., Zhou Y.F., Yu J., Li X.X., Zhang N. (2023). SEPT2 crotonylation promotes metastasis and recurrence in hepatocellular carcinoma and is associated with poor survival. Cell Biosci..

[102] Kinoshita M., Kumar S., Mizoguchi A., Ide C., Kinoshita A., Haraguchi T., Hiraoka Y., Noda M. (1997). Nedd5, a mammalian septin, is a novel cytoskeletal component interacting with actin-based structures. Genes Dev..

[103] Silió V., Marqués M., Cortés I., Zuluaga S., Carrera A.C. (2007). A cascade involving p85, Cdc42 and septin 2 regulates cytokinesis. Biochem. Soc. Trans..

[104] Sun L., Li Y., Wang M., Luo L., Sun R., Chen Y., Bai Y., Ding C., Wang Y. (2024). p53 deficiency mediates cisplatin resistance by upregulating RRM2 and crotonylation of RRM2(K283) through the downregulation of SIRT7. Front. Mol. Biosci..

[105] Siegel R.L., Miller K.D., Fuchs H.E., Jemal A. (2021). Cancer Statistics, 2021. CA Cancer J. Clin..

[106] Wei W., Zeng H., Zheng R., Zhang S., An L., Chen R., Wang S., Sun K., Matsuda T., Bray F. (2020). Cancer registration in China and its role in cancer prevention and control. Lancet Oncol..

[107] Fu Q.F., Liu Y., Fan Y., Hua S.N., Qu H.Y., Dong S.W., Li R.L., Zhao M.Y., Zhen Y., Yu X.L. (2015). Alpha-enolase promotes cell glycolysis, growth, migration, and invasion in non-small cell lung cancer through FAK-mediated PI3K/AKT pathway. J. Hematol. Oncol..

[108] Cheng Z., Shao X., Xu M., Zhou C., Wang J. (2019). ENO1 Acts as a Prognostic Biomarker Candidate and Promotes Tumor Growth and Migration Ability Through the Regulation of Rab1A in Colorectal Cancer. Cancer Manag. Res..

[109] Yu H., Bu C., Liu Y., Gong T., Liu X., Liu S., Peng X., Zhang W., Peng Y., Yang J. (2020). Global crotonylome reveals CDYL-regulated RPA1 crotonylation in homologous recombination-mediated DNA repair. Sci. Adv..

[110] Abu-Zhayia E.R., Machour F.E., Ayoub N. (2019). HDAC-dependent decrease in histone crotonylation during DNA damage. J. Mol. Cell Biol..

[111] Wang Y., Dong T., Wang P., Li S., Wu G., Zhou J., Wang Z. (2021). LINC00922 regulates epithelial-mesenchymal transition, invasive and migratory capacities in breast cancer through promoting NKD2 methylation. Cell. Signal..

[112] Liao M., Sun X., Zheng W., Wu M., Wang Y., Yao J., Ma Y., Gao S., Pei D. (2023). LINC00922 decoys SIRT3 to facilitate the metastasis of colorectal cancer through up-regulation the H3K27 crotonylation of ETS1 promoter. Mol. Cancer.

[113] Hou J.Y., Gao L.J., Shen J., Zhou L., Shi J.Y., Sun T., Hao S.L., Wang D.P., Cao J.M. (2023). Crotonylation of PRKACA enhances PKA activity and promotes colorectal cancer development via the PKA-FAK-AKT pathway. Genes Dis..

[114] Liu N., Konuma T., Sharma R., Wang D., Zhao N., Cao L., Ju Y., Liu D., Wang S., Bosch A. (2023). Histone H3 lysine 27 crotonylation mediates gene transcriptional repression in chromatin. Mol. Cell.

[115] Sundström P., Ahlmanner F., Akéus P., Sundquist M., Alsén S., Yrlid U., Börjesson L., Sjöling Å., Gustavsson B., Wong S.B. (2015). Human Mucosa-Associated Invariant T Cells Accumulate in Colon Adenocarcinomas but Produce Reduced Amounts of IFN-γ. J. Immunol..

[116] Won E.J., Ju J.K., Cho Y.N., Jin H.M., Park K.J., Kim T.J., Kwon Y.S., Kee H.J., Kim J.C., Kee S.J. (2016). Clinical relevance of circulating mucosal-associated invariant T cell levels and their anti-cancer activity in patients with mucosal-associated cancer. Oncotarget.

[117] Hou J.Y., Li N., Wang J., Gao L.J., Chang J.S., Cao J.M. (2023). Histone crotonylation of peripheral blood mononuclear cells is a potential biomarker for diagnosis of colorectal cancer. Epigenetics Chromatin.

[118] Guo Z., Zhang Y., Wang H., Liao L., Ma L., Zhao Y., Yang R., Li X., Niu J., Chu Q. (2024). Hypoxia-induced downregulation of PGK1 crotonylation promotes tumorigenesis by coordinating glycolysis and the TCA cycle. Nat. Commun..

[119] Ju Z., He J.J. (2017). Prediction of lysine crotonylation sites by incorporating the composition of k-spaced amino acid pairs into Chou’s general PseAAC. J. Mol. Graph. Model..

[120] Bos J., Muir T.W. (2018). A Chemical Probe for Protein Crotonylation. J. Am. Chem. Soc..

[121] Malebary S.J., Rehman M.S.U., Khan Y.D. (2019). iCrotoK-PseAAC: Identify lysine crotonylation sites by blending position relative statistical features according to the Chou’s 5-step rule. PLoS ONE.

[122] Spinck M., Neumann-Staubitz P., Ecke M., Gasper R., Neumann H. (2020). Evolved, Selective Erasers of Distinct Lysine Acylations. Angew. Chem. Int. Ed. Engl..

[123] Xie Y., Yang L., Chen Q., Zhang J., Feng L., Chen J.L., Hao Q., Zhang L., Sun H. (2021). Single-step fluorescent probes to detect decrotonylation activity of HDACs through intramolecular reactions. Eur. J. Med. Chem..

[124] Lv H., Dao F.Y., Guan Z.X., Yang H., Li Y.W., Lin H. (2021). Deep-Kcr: Accurate detection of lysine crotonylation sites using deep learning method. Brief. Bioinform..

[125] He J., Wang S., Liu X., Lin R., Deng F., Jia Z., Zhang C., Li Z., Zhu H., Tang L. (2020). Synthesis and Biological Evaluation of HDAC Inhibitors With a Novel Zinc Binding Group. Front. Chem..

[126] Hou J.Y., Zhou L., Li J.L., Wang D.P., Cao J.M. (2021). Emerging roles of non-histone protein crotonylation in biomedicine. Cell Biosci..

